# Supplementary material for: Quantifying the effects of antibiotic resistance and within-host competition on strain fitness in Streptococcus pneumoniae
Source: PLoS Biol. 2025 Aug 5;23(8):e3003300. doi: 10.1371/journal.pbio.3003300 (PMC12324126; doi:10.1371/journal.pbio.3003300)
Supplement: Attachment — Submitted filename: Response_to_reviewers.pdf [file pbio.3003300.s002.pdf]

Response to Reviewers: Quantifying the effects of antibiotic resistance and within-host competition on strain fitness in *Streptococcus pneumoniae*

Krishna et. al. 2025

June 6, 2025

We thank the editor and reviewers for their thoughtful reviews and helpful feedback. We have revised the manuscript according to the comments; please find our detailed responses below.

1 Reviewer 1

In this manuscript, Krishna et al. use a large longitudinal dataset of infants colonized with different strains of S. pneumoniae, with varying levels of antibiotic resistance. Using statistical models, they make inferences about the degree of competition among strains, that is to say how the presence of one strain affects the time to clearance or colonization by other strains, and how such effects vary depending on antibiotic treatments and resistant bacteria. The combination of a unique dataset, thoughtful analysis, and clear writing in the context of the literature make this an important and interesting contribution. The authors clearly show large effects of within-host competition increasing clearance rates and reducing establishment of incoming strains. By contrast, they find smaller effects of antibiotic resistance and relatively small fitness costs of resistance. Counter-intuitively, they find a negative association between a strain's inherent rate of clearance and its change in clearance rate due to competition. This suggests an evolutionary tradeoff between colonization ability and competitive ability - a hypothesis that could be tested with future datasets and experiments.

In general, the analyses are well-explained and the conclusions are well-supported, with appropriate caveats. I am not familiar with Cox hazard models, but these are well-explained and well-justified, and use permutation tests are done to account for possible confounds.

Response: We thank the reviewer for the positive feedback.

Specific comments:

1) I do have some questions about the underlying data: can we be sure that strain diversity is exhaustively sampled, and does sampling effort vary over time? Are the analyses sensitive to assumptions about sampling completeness, i.e. if diversity is undersampled (or if certain strains are undersampled, etc) would this bias the results in any way? A bit more explanation of the sampling methods, and perhaps something akin to collector's curves (or rarefaction analysis) showing the completeness of sampling would be useful.

Response: The reviewer has raised valid points about the effects of sampling on our results. While sampling efforts have not varied over time, sampling cover is not exhaustive in the dataset we use. There are two components of sampling completeness to be noted here - (A) not all infants in the Maela camp have been sampled, and this might lead to some serotypes missed from the dataset; and (B) in a given sample, the serotyping methods used do not fully capture the within-host diversity in the host [1, 2], and this might lead to low frequency serotypes in the host being missed.

In the case of (A), we think that our dataset is a representative sample of the Maela camp since around 12% of infants in the camp (added at line 712-713) were recruited in the sampling study. We have now added a rarefaction curve in Supplementary Figure S1D and introduced it at line 720. We find that the diversity captured with our data only increases weakly with sample size after around 200 infants are sampled; at that point, the dataset always captures at least 60 out of the 68 serotypes that we observe, suggesting only rare serotypes would be missed with our sample size.

In the case of (B), undersampling of within-host diversity could affect the results on how within-host competitors affect clearance or establishment. To test the robustness of our results to this effect, we removed observed strains from samples with a probability ranging from 1% to 50% (per strain, per time-point) to simulate undersampling. We repeat our analyses under these undersampling regimes and find that our main results are qualitatively robust. The exception is the priority effect we detect: here, undersampling exaggerates the difference between invader and resident competitors. This reduces our confidence in the priority effect as it could be driven by undersampling. We now discuss the above in the Methods (lines 761-769) and Limitations (lines 687-696) sections, and in Supplementary Note 3 (line 1100) and Supplementary Figure S4.

2) A related question: it is implied that each strain (e.g. 19F) is matched with a resistance phenotype (e.g. 19F-R and 19F-S) but it is unclear if this is the case, or if resistance phenotypes are not linked to serotype identity. Please clarify.

Response: Yes, this is correct. To be more precise, each episode of carriage is defined based on serotype (eg. 19F). Whenever resistance is also measured, the episode of carriage can additionally be labelled as resistant (19F-R) or a susceptible (19F-S) to a given antibiotic.

To clarify this better in the manuscript, we have added the following sentence along with further clarification in the main text at lines 775-778: "... an episode of carriage defined by its serotype has a constant resistance status - for instance '6B-resistant' or '6B-susceptible' - when resistance is known.".

3) There is an implicit assumption that resistance or sensitivity does not evolve within hosts, but is there justification for this assumption - either from the literature or based on this dataset? In principle, it seems possible that antibiotic treatment could actually select for de novo resistance within hosts, which would be observed as a switch of S to R without a change in serotype. Does this occur? Maybe it is negligible, but it is a hypothesis that could be tested, which could also help explain some of the variation in resistance over time and across patients.

Response: We thank the reviewer for pointing this out, this is indeed possible. To quantify how often this occurs, we looked at episodes of carriage where resistance phenotypes were measured more than once. Across all episodes of carriage where resistance was known (across all antibiotics), discordant resistance estimation was observed in 1.9% of cases (0.9% cases are S$\rightarrow$R changes and 1% are R$\rightarrow$S changes). This corresponds to 4.5% of episodes of carriage during which resistance was measured multiple times. It is possible that these cases could correspond to within-host acquisition or loss of resistance.

We repeated our results on antibiotic consumption and resistance by removing the episodes of carriage (the 1.9%) where resistance might have been gained or lost, and do not find qualitative changes in our results. We have added this and further clarifications on how we deal with resistance data in the main text (lines 774-788), included the sensitivity test in Supplementary Note 8 (line 1200) and added a corresponding Supplementary Figure S11.

4) A related question is whether there could be within-host serotype switches, or if this is known from the literature to be negligible.

Response: Serotype switching is indeed possible, but rare. A previous meta-analysis of four studies gives an estimate for the rate of serotype switching at 1.5 x 10^{-3} per week per strain in *S. pneumoniae* [3]. If we assume this to be a uniform rate across all serotypes and that it scales linearly, this rate would translate to around 64 expected serotype switch events across 5529 episodes of carriage. We therefore assume that the effect of serotype switching on our results is negligible.

We have now added a sentence in our main text explicitly stating this assumption in lines 759-760.

5) Finally, a semantic distinction that I think is important to clarify the hypothesis being tested: in the title, abstract, and throughout, the authors implicitly assume that strains (serotypes) compete, and seek to quantify the strength of this competition. But the paper could be better framed as asking whether competition occurs, and how to quantify it. I think that a neutral model of strains with equal fitness colonization at random is effectively the null hypothesis being considered, but the title and abstract take it almost for granted the competition does occur. I suggest modifying the title to something more like: "Quantifying the effects of antibiotic resistance and within-host competition in Streptococcus pneumoniae." (cutting the term 'fitness' because it is effectively synonymous with competition in the context of this study).

Response: We thank the reviewer for bringing up this point. We agree the framing implicitly assumes competitive interactions between strains and that it is helpful to contrast this with a neutral model and clarify how this relates to the statistical analysis. Our implicit assumption that interactions are competitive was based on previous studies. This was not made clear in the previous version of the manuscript.

We have edited the introduction to better frame competition in contrast to a neutral model and to better contextualise why we frame the work in terms of quantifying effect sizes rather than hypothesis-testing (lines 40-49). We have clarified the text in relation to the introduction of the '*presence-of-competitor*' variables (lines 200-205). We have also clarified the relationship between the statistical analysis and neutral model (lines 216-218).

We would prefer to keep the title as it is because while we agree with the reviewer "quantifying the effects of within-host competition" is equivalent to (and more succinct than) our original version, we find "quantifying the effects of antibiotic resistance in *Streptococcus pneumoniae*" (without the "on fitness" part) too unspecific.

2 Reviewer 2

In this paper, Krishna et al. explore competition dynamics within the host for Streptococcus pneumoniae, using one of the largest infant nasopharyngeal swab datasets available. By applying Cox Proportional Hazards models across multiple independent frameworks, the authors provide a quantitative view of strain clearance and establishment rates. Their results suggest that intra-host competition likely plays a key role in pneumococcal persistence, and they also raise interesting points about the potential roles of priority effects and antibiotic resistance genes in shaping strain fitness before and after antibiotic treatment.

As the authors note in the discussion, studies in E. coli and even earlier S. pneumoniae work (e.g., Lourenço et al., Vaccines 2021; Trzciński et al., mBio 2015) have tackled similar questions. However, what sets this study apart is the strength of the statistical modeling and the ability to make chronological inferences with higher resolution. The study is engaging, methodologically solid, and I believe it makes a valuable contribution that is well-suited for PLOS Biology.

Response: We thank the reviewer for this positive feedback.

I have only a few minor comments, aimed mainly at sharpening the manuscript further:

Results:

* Please make sure all supplementary figures are clearly and consistently annotated — this will help readers navigate the data more easily.

Response: Thanks for pointing this out. We have added sub-figure titles (Figures S1, S5, S7), adjusted axis labels (Figures S3, S7, S8, S10), and modified captions/caption titles where necessary (Figures S1, S3, S5, S6, S7), to improve the readability of all supplementary figures.

* Lines 189-195: Please rephrase this section to: "The exponential of the hazard coefficient yields a hazard ratio of 1.62 (95% CI: 1.45-1.80), indicating that antibiotic consumption increases the hazard

of strain clearance by 62%." I recommend applying this style consistently across the paper. Hazard ratios should be reported directly with their confidence intervals, not as percentage increases, to avoid confusion.

Response: We have rephrased the sentence as suggested. We have also provided hazard ratios and their confidence intervals wherever reported in the manuscript. Changes have been made at line numbers 191-194, 220, 381, 385, 446-450, 458-459, 479, 487-488, 497-498, and 1140-1141.

* On p-values: if you present absolute p-values in some places (e.g., lines 334 and 275), please keep that approach consistent throughout the manuscript.

Response: Thanks for picking up on this inconsistency. In principle, we prefer to report absolute p-values as they contain more information, and this is the format we mostly use (including throughout the supplement). However, on line 275, we report p <0.01 because this is based on a permutation test with 100 iterations, with no value in the simulated distribution greater than the observed value. We are therefore unable to estimate an absolute p-value here.

Discussion:

* It would be helpful if the authors briefly explain why they chose the Cox model over other options like Accelerated Failure Time (AFT) models or flexible parametric models. Even a short rationale would add transparency and show that the choice was deliberate, not default.

Response: We have added a short text justifying this choice in lines 813-820.

* I also recommend adding a short "Limitations" section. Some of the findings, especially regarding priority effects, could be quite sensitive to how colonization episodes are defined. Flagging this explicitly would help readers interpret the results with the right level of caution and would strengthen the overall credibility of the paper.

Response: Good suggestion, we have now included a Limitations paragraph in the Discussion at line 667.

3 Other changes

We have made some other minor corrections to the text:

1. In the caption for Figure 5D, the letter C and changed to B. In line 1183, "Figure SS-" was replaced with "Figure S-" These were typographical errors.

2. We made corrections to spellings to make them consistent with UK English, for example colonize to colonise, or summarize to summarise. These are in lines 169, 1091, 1216 and captions for Figures 1, S2, S3, S7.

3. We corrected the number of serotypes which was incorrectly mentioned earlier in lines 132, 717-719 and 831-832: in the whole dataset, 72 serotypes were identified across mothers and infants (excluding non-typeables), while 68 serotypes were identified in infants alone.

4. In lines 895-897, we have added a legend for the Supplementary information associated with the main text.

References

[1] P Turner, et al., Field Evaluation of Culture plus Latex Sweep Serotyping for Detection of Multiple Pneumococcal Serotype Colonisation in Infants and Young Children. *PLoS ONE* **8**, e67933 (2013).

[2] G Tonkin-Hill, et al., Pneumococcal within-host diversity during colonization, transmission and treatment. *Nature Microbiology* **7**, 1791–1804 (2022) Publisher: Nature Publishing Group.

[3] L Temime, PY Boelle, L Opatowski, D Guillemot, Impact of Capsular Switch on Invasive Pneumococcal Disease Incidence in a Vaccinated Population. *PLOS ONE* **3**, e3244 (2008) Publisher: Public Library of Science.
